# Prediction of the Work-Hardening Exponent for 3104 Aluminum Sheets with Different Grain Sizes

**DOI:** 10.3390/ma12152368

**Published:** 2019-07-25

**Authors:** Ni Tian, Fei Yuan, Ceheng Duan, Kun Liu, Guangdong Wang, Gang Zhao, Liang Zuo

**Affiliations:** 1 Key Laboratory for Anisotropy and Texture of Materials, School of Materials Science &Engineering, Northeastern University, Shenyang 110819, China; 2 Research Center for Metallic Wires, Northeastern University, Shenyang 110819, China; 3 Department of Applied Science, University of Quebec at Chicoutimi, Chicoutimi, QC G7H2B1, Canada

**Keywords:** annealed 3104 aluminum sheet, grain size, *n* value, target strain, yield strength

## Abstract

A practical approach to predict the yield strength and work-hardening exponent (*n* value) to evaluate the deep-drawing performance of annealed 3104 aluminum sheets is presented in the present work by only measuring and analyzing the grain size of the sheet. The various grain sizes were obtained through the different annealing treatment and then the evolution of the *n* value under different strains and the yield strength of annealed 3104 aluminum sheet were evaluated. Results showed that the *n* value and yield strength vary greatly with the grain size. A mathematical model relating grain size *d*, work-hardening exponent *n*, target strain *ε*, and yield strength *R*_p0.2_ was developed in the present work. Within the studied grain size range *d* (12–29 μm), the *n* value generally increased with *d* in a strain-dependent manner, such that n=0.1875−85.03 × exp[−d/1.94] when the *ε* was less than 0.5%, but $n=0.3-0.15d^{-1/2}$ when the *ε* was greater than 2%. On the other hand, the *n* value was found to depend on the target strain *ε* as $n=0.276-A_{1}\times \exp\left[-e/1.0435\right]$, where *A*_1_ varies with *d* and its value is in the range of 0.132–0.364. In addition, the relationship between *R*_p0.2_ and *d* followed the Hall-Petch equation ($R_{p0.2}=36.273\;+\;139.8\;\times \;d^{-1/2}$).

## 1. Introduction

Both the work-hardening exponent (*n* value) and the yield strength are the essential parameters governing the deep-drawing properties of sheets. In general, for most metallic material sheets, the higher the *n* value is, the lower the yield strength is, and the better the deep-drawing property is. The *n* value and yield strength are strongly dependent on the microstructure of the sheet, such as grain size, second-phase particles, dislocation density, etc. Researchers have attempted to reveal the relationship between microstructures and *n* value as well as yield strength in order to predict the deep-drawing properties of sheets. It is well-known that the relationship between yield stress and grain size of most metals and alloys can be expressed by the Hall-Petch equation though with different Hall-Petch constants from the different materials or microstructures [1,2]. However, there are few studies about the relationship between grain size *d*, calculated strain point, and the work-hardening exponent *n* [3,4,5]. Previously [6], it was observed that the *n* value for Al-Mg-Si-Cu alloy sheets initially increased before stabilizing with increasing strain in a solid solution and in T4 states; on the other hand, after annealing, it initially increased and then decreased under the same conditions. These different behaviors can be attributed to the various microstructures resulting from the different conditions, especially the annealing condition. The *n* value was also reported to differ in different alloys subjected to the same strain conditions. Liu et al. [7] found that *n* was greatest for AA5056, followed by AA2024, AA2014, and AA7178 under identical strain conditions, although *n* consistently increased with strain in all cases. Mo et al. [8] reported that *n* for A319-T6 aluminum alloy is quite sensitive to the secondary dendrite arm spacing (SDAS) and Si particles (e.g., their aspect ratio and volume fraction). The *n* value decreases with increasing of SDAS and aspect ratio of Si particles. Tsuchida [9] reported that *n* of industrial pure aluminum is also related to dislocation dynamics. Many studies have also shown that the distributions of intermetallics and dislocations significantly affect *n* in metals and alloys [10,11,12,13]. The *n* value is thus clearly influenced by distribution of intermetallics and particles, as well as by dislocations. However, there is scant literature on the impact of grain size *d* on *n* value in aluminum alloys.

Some researchers have worked out the relationship between *n* value and the grain size *d* for steel [14,15,16], which is convenient to predict the deep-drawing ability of steel. Morrison [14] found that *n* increases with *d* in low-carbon steel, according to the empirical formula $n=\frac{5}{10+d^{-1/2}}$ (*d* in mm). After fully considering the comprehensive interactions between *d*, the size and volume of precipitates, and the dislocation density in Ti-IF steel, Antoine [15] proposed a model for predicting *n* as

(1) $$n=0.450-0.001\left[\sigma_{\mathrm{eff}}+\frac{6.6}{\sqrt{d}}+\sum_{\mathrm{TiX}}\left(0.00065\frac{f^{1/2}{{}_{V}}_{{}_{\mathrm{TiX}}}}{{\bar{r}}_{\mathrm{TiX}}}\right)\times \ln\left(\frac{{\bar{r}}_{\mathrm{TiX}}}{2.48\times 10^{-7}}\right)+1.92\times 10^{-2}\sqrt{\rho_{F0.2}^{}}\right]$$

where, $\sigma_{\mathrm{eff}}$ is a short distance interaction stress or effective stress, *d* is the grain size (mm), $f_{V_{\mathrm{TiX}}}$ and ${\bar{r}}_{\mathrm{TiX}}$ are the volume fraction and average radius of the TiX-type precipitates, respectively, and $\rho_{F0.2}$ is the dislocation density at the yield strength. It can be found that the models proposed by Morrison and Antoine for calculating *n* in steel are significantly different, despite low-carbon steel and Ti-IF steel both displaying a body-centered cubic (BCC) structure. Qiu [16] found that *n* linearly increases with decreasing ferrite grain size *d*^−1/2^, and then proposed a model for predicting *n* as $n=0.3301-0.2401d^{-1/2}$ (*d* in the range of 1.30~37 μm) in ultrafine-grained steels. Gashti [17] found that AA1050 aluminum alloy sheets show lower *n* due to grain refinement.

On the other hand, there are presently no established models for aluminum alloys and therefore a strong motivation in industry for developing a model to predict *n* for a deep-drawn aluminum sheet. Therefore, the non-age-hardening 3104 aluminum alloy, which is widely used in industry for fabricating deep-drawing aluminum sheets, was investigated to determine its relationship between *n* value, yield strength, and grain size obtained from various annealing processes. The mathematical model was established based on the experimental data, aiming to predict the *n* value for annealed 3104 aluminum sheets and then provide experimental data for the stamping process of 3104 aluminum alloy sheets or other aluminum alloy sheets.

## 2. Materials and Methods

**Figure 1:**
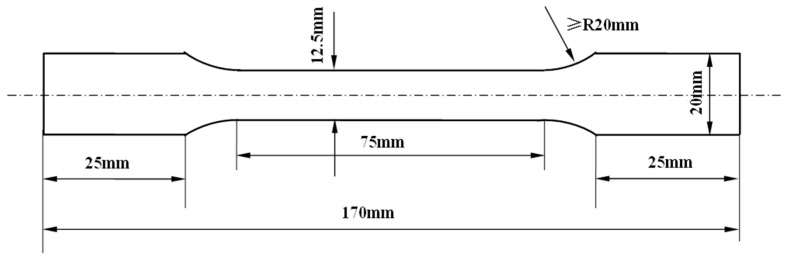
Dimensions of the tensile specimens.

Semicontinuous DC (direct chill) casting of 3104 aluminum alloy was performed in the lab with the dimensions of 200 mm (width) × 80 mm (thickness) × 600 mm (length) with the composition of (wt%) 1.21% Mn, 1.04% Mg, 0.25% Fe, 0.30% Si, 0.18% Cu, 0.11% Ti, and Al balance. The specimen for the chemical composition analysis was cut from the casting ingot and analyzed by an Oxford Foundry-Master Pro Optical Emission Spectrometer (Oxford Instruments PLC, Uedem, Germany). The ingot was homogenized at 580 °C for 24 h, followed by hot-rolling at 480 °C to 6 mm, and then cold-rolled to 1.0 mm sheet. After rolling, sheets were annealed at 400 °C/1 h, 500 °C/1 h, 550 °C/1 h, 4 h, 8 h, and 24 h to obtain different grain sizes. After each annealing process, three specimens were cut from the sheet along the rolling direction and then machined to the dimensions in Figure 1 according to ASTM (American Society for Testing Material) E8/E8M-2013a for tensile testing. Tensile tests were conducted using a Shimadzu AG-X100KN electronic universal testing machine (Shimadzu Coporation, Kyoto, Japan) at room temperature with a strain rate of 0.08 min^−1^. The true stress vs. true strain curve was drawn by determining the real-time load and the real-time geometry size of specimen (the real-time length/width), which were measured by a load sensor, a length extensometer with 50 mm gauge length, and a width extensometer with 12.5 mm gauge length, respectively. Specimens for metallography were cut from the tensile specimens before tensile deformation and then ground with sandpaper till #5000 and finalized with the fine polishing on the polishing machine. The morphology and distribution of the intermetallics and dispersoids after each annealing treatment were observed using an Olympus GX71 optical microscope (OM) (Olympus Coporation, Tokyo, Japan) and a Tecnai G2 transmission electron microscope (TEM) (FEI Company, Hillsboro, OR, USA). TEM samples were cut from the tensile specimen before the tensile deformation, mechanical-thinned to approximately 90 μm, and then electropolished by twin-jet in a solution of 25% HNO_3_ + 75% CH_3_OH at −25 °C and under a voltage of 15 V (MTP-1A magnetic twin-jet electropolishing, Shanghai Jiaoda electromechanical technology development Co. Ltd., Shanghai, China). The grain size was revealed by electropolishing in a solution of 25% HNO_3_ + 75% CH_3_OH at −5 °C and under a voltage of 20 V and characterized by electron back-scatter diffraction (EBSD) with a step size of 2 μm and 40 kV acceleration voltage by a JMS-7001F scanning electron microscope (SEM) (JEOL Ltd., Tokyo, Japan).

The true stress and strain of the aluminum sheet are assumed to obey $\sigma =K\varepsilon^{n}$ or, equivalently, logσ=logK+nlogε. The *n* value was therefore calculated for different sheet strains by a least-squares approach:

(2) $$n=\frac{N\sum_{i=1}^{N}\log\varepsilon_{i}\cdot \log\sigma_{i}-\sum_{i=1}^{N}\log\varepsilon_{i}\cdot \log\sigma_{i}}{N\sum_{i=1}^{N}{\left(\log\varepsilon_{i}\right)}^{2}-{\left(\sum_{i=1}^{N}\log\varepsilon_{i}\right)}^{2}}$$

where *N* = 20 is the number of strain points in the target strain range, and ε*_i_* and σ*_i_* are the instantaneous true strain and stress corresponding to the calculated strain point, respectively. The *n* value was determined by increasing the strain within the uniform plastic deformation range, starting from the yield point ε = 0.2%.

## 3. Results and Discussion

### 3.1. Microstructures of 3104 Sheets after Various Annealing Treatments

**Figure 2:**
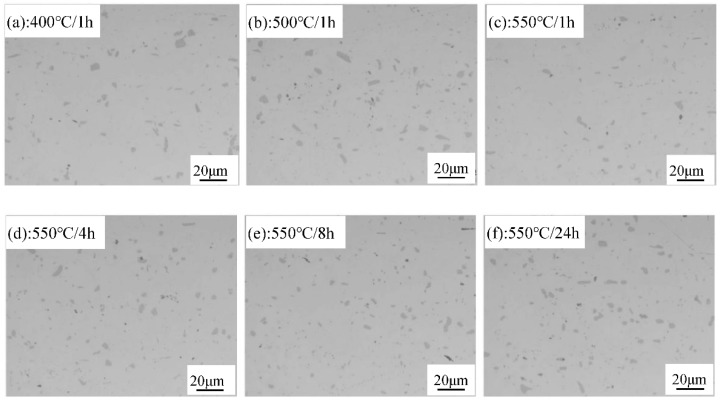
Microstructure of 3104 alloy sheet after various annealing treatments: (**a**) 400 °C/1 h; (**b**) 500 °C/1 h; (**c**) 550 °C/1 h; (**d**) 550 °C/4 h; (**e**) 550 °C/8 h; (**f**) 550 °C/24 h.

**Figure 3:**
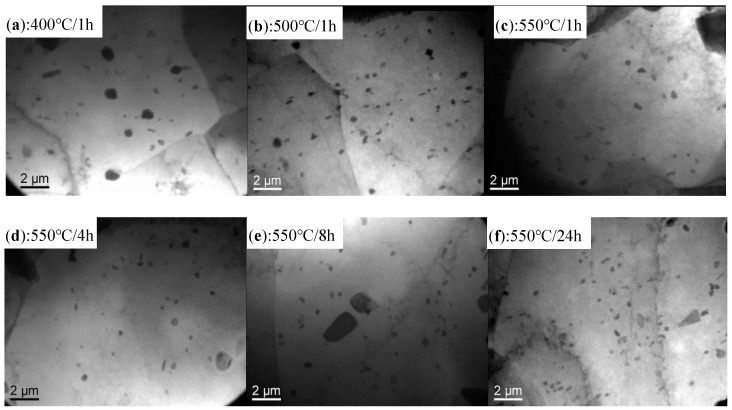
TEM images of 3104 alloy sheets after various annealing treatments: (**a**) 400 °C/1 h; (**b**) 500 °C /1 h; (**c**) 550 °C/1 h; (**d**) 550 °C/4 h; (**e**) 550 °C/8 h; (**f**) 550 °C/24 h.

OM and TEM observations of 3104 sheets after various annealing treatments are displayed in Figure 2 and Figure 3, respectively. There are many broken micron-scale intermetallics in Figure 2, while some fine particles (~200 nm) are observed in Figure 3. According to the SEM-EDS results and the literature [18], the broken intermetallics consist of Al_6_(MnFe), which is formed during solidification and then fragmented into small particles during the rolling process. The high melting point of these intermetallics prevents their dissolution into the matrix during annealing, so they remain embedded within the matrix [18]. As shown in Figure 2, the size and volume of the Al_6_(MnFe) particles are relatively independent of the applied annealing protocol, as they are closely related to the Mn and Fe contents in the alloy [18]. Some fine particles, reportedly α-Al(MnFe)Si dispersoids, are also observed in the TEM image of Figure 3 [18]. Their volume fraction is relatively low and their size is consistently within the range of 100–200 nm regardless of the annealing protocol. Therefore, in summary, the intermetallic and dispersoid characteristics (volume, size, and distribution) are relatively independent of the precise annealing protocol.

**Figure 4:**
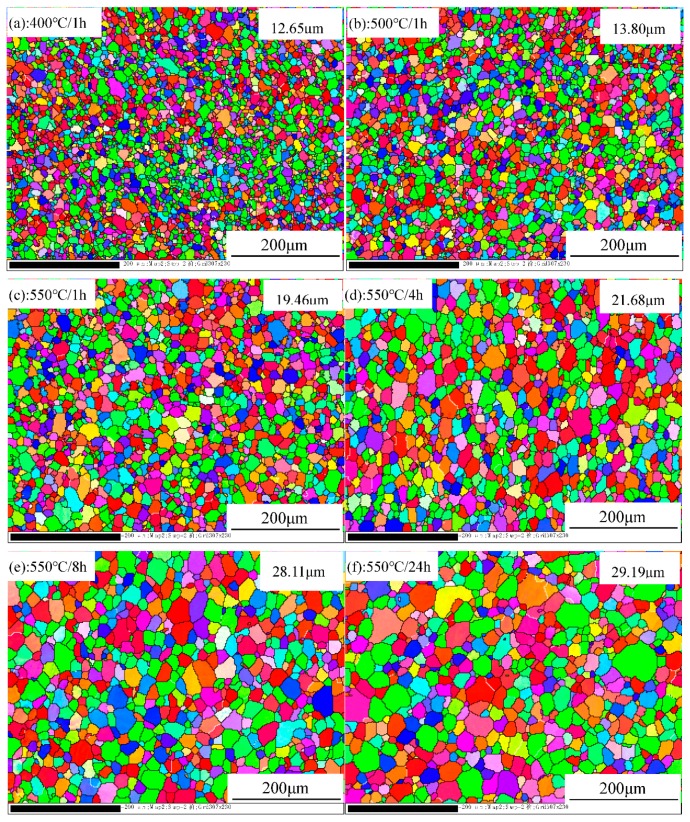

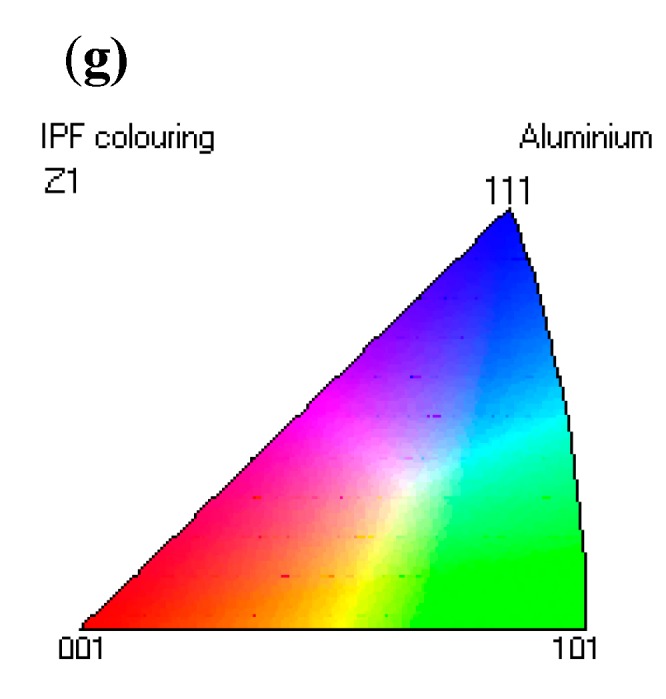
EBSD mapping of 3104 sheets after various annealing treatments: (**a**) 400 °C/1 h; (**b**) 500 °C/1 h; (**c**) 550 °C/1 h; (**d**) 550 °C/4 h; (**e**) 550 °C/8 h; (**f**) 550 °C/24 h; (**g**) orientation triangle for EBSD mapping

Figure 4 shows the EBSD results after various annealing processes. Full recrystallization is achieved under all annealing conditions, as evidenced by the uniform equixed grains with random orientations, and without any texture. However, the measured grain size varies with the annealing process, increasing from 12.65 μm after 400 °C/1 h to 13.80 μm after 500 °C/1 h, 19.46 μm after 550 °C/1 h, 21.68 μm after 550 °C/4 h, 28.11 μm after 550°C/8 h, and 29.19 μm after 550 °C/24 h. The increase in grain size can be attributed to the increase either in the annealing temperature or the holding time. As shown in Figure 4, full recrystallization is already achieved after only 400 °C/1 h annealing. The grain therefore begins to grow with increasing annealing temperature and holding time [19,20]. Hence, the specimens with different grain sizes and without any texture but similar distributions of intermetallics and dispersoids in the matrix were obtained by controlling the annealing process, providing the premise to study the effect of the grain size on their properties and the *n* value.

### 3.2. Dependence of the Yield Strength on Grain Size

**Figure 5:**
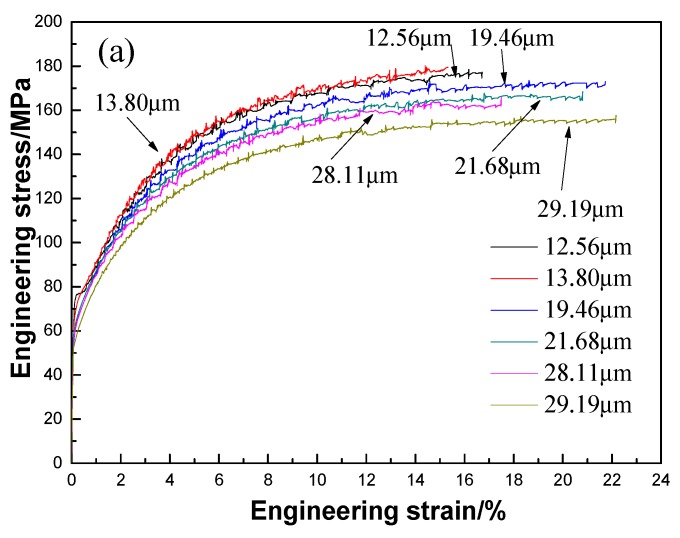

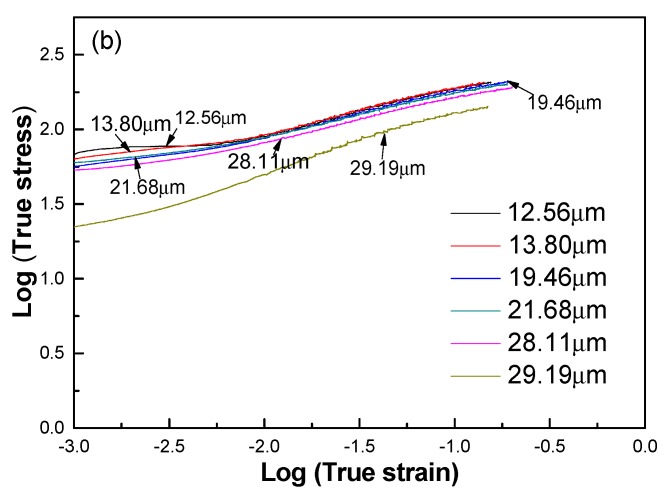
Complete typical engineering strain-engineering stress (**a**) and uniform plastic deformation part of log (true strain)-log (true stress) (**b**) curves of 3104 sheets with different grain size.

Figure 5 plots the typical engineering strain versus engineering stress and log (true strain) versus log (true stress) for 3104 alloy sheets after various annealing treatments during the tensile test. Serrated flow is apparent in Figure 5 for all the annealed sheets, a phenomenon referred to as the Portevin-LeChatelier (PLC) effect [21,22], which may be attributed to the dynamic strain aging (DSA) that occurs during tensile deformation [23,24]. During the rolling process and the subsequent annealing, the intermetallics are fragmented into small particles and dispersoids are precipitated in the matrix, both of which can hinder the movement of dislocations. Meanwhile, as shown in Figure 2 and Figure 3, the spacings between particles and dispersoids are relatively large and therefore dislocations can slip between them without hindrance until they become pinned by an encountered particle or dispersoid. This effect gives rise to the serrated flow stress observed in the stress-strain curves of Figure 5. This phenomenon was also reported in annealed 5082 aluminum alloy by Abbadi et al. [25]. However, PLC characteristics (e.g., critical strain and the strain decrease) are similar under all the annealing conditions, reflecting the weak influence of the grain size on PLC behavior. This can be explained mainly in terms of similarities in the microstructure, in particular, the distribution of particles and dispersoids in annealed 3104 alloy sheets. As shown in Figure 2 and Figure 3, the size, volume fraction, and distribution of particles and dispersoids are similar after various annealing treatments. Therefore, this gives rise to the similar PLC behavior despite the different grain sizes.

**Figure 6:**
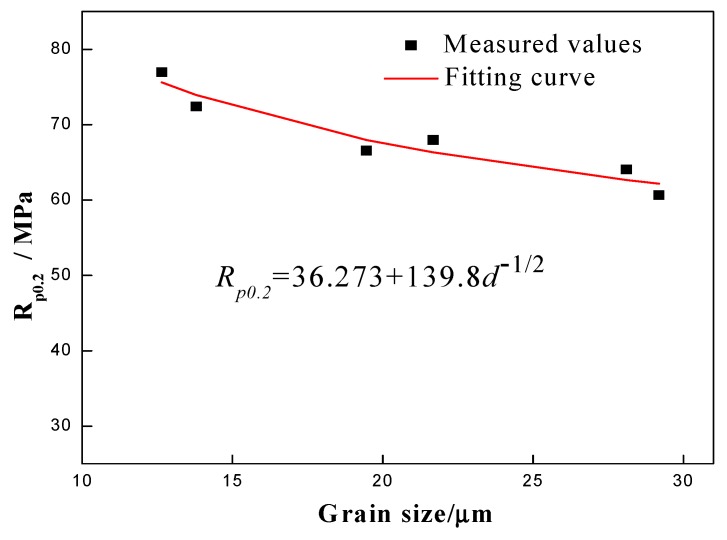
Relationship between grain size and yield strength and its fitting result.

Figure 5 also shows the significant dependence of the flow stress on the grain size. Figure 6 summarizes the relationship between grain size and yield strength and its fitting results. It can be seen that the yield strengths decrease slowly for grain sizes up to 29.19 μm. The fit of the grain size data to the yield strength is consistent with the Hall-Petch law as $R_{p0.2}=36.273+139.8\times d^{-1/2}$ for studied grain sizes in the range of 12.65–29.19 μm.

### 3.3. Dependence of the Strengthen-Hardening Exponent (n Value) on Grain Size

**Figure 7:**
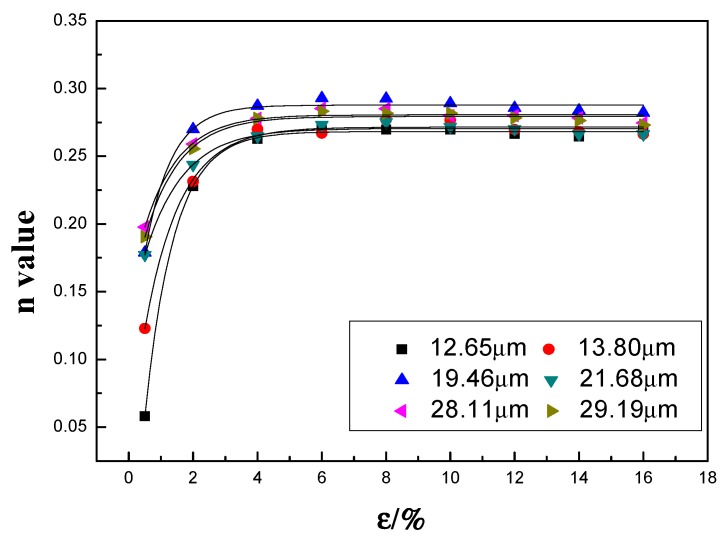
Evolution of *n* value at different target strains and the fitting results.

Figure 7 shows the dependence of *n* on the target strains for different grain sizes. An initial rise in *n* is generally observed for strains under 2%, followed by a slow increase between 2% and 4%, and then a slight decrease beyond 4%.

As shown in Figure 2 and Figure 3, the matrix contains a large volume of fragmented particles and dispersoids that can pin the dislocations. However, these particles and dispersoids are relatively large (in the order of microns) and dislocations can readily pass them via the Orowan bypass mechanism and then leave one dislocation loop, thereby reducing the effective particle spacing. It is therefore increasingly difficult for subsequent dislocations to pass in the same manner, which leads to a rapid increase in *n* at lower stains in the initial stage of tensile deformation (~2% in the present study). A similar phenomenon was reported in annealed Al-Mg-Si sheets [6]. For strains beyond 2%, a stronger resistance to dislocation motion is expected because of the activated multi-slipping system and the intersection between dislocations form jogs and Lomer-Cottrell dislocation locks. However, flow stress increases rapidly because of the large *n* value, making it possible for edge dislocations to climb around and for screw dislocations to cross-slip obstacles. Hence, *n* displays a downward trend. The dependence of *n* on the target strain *ε* during deformation obeys the following equation:

(3) $$n=A_{0}-A_{1}\times \exp[-\varepsilon /t]$$

where *A*_0_, *A*_1_, and *t* are calculated and summarized in Table 1. It can be found that *A*_0_ and *t* are in the range of 0.268~0.288 and 0.820~1.192, respectively, while *A*_1_ is changing from 0.132–0.364. It can be approximately calculated that both *A*_0_ and *t* are constant, and the weighted average value of *A*_0_ and *t* are 0.276 and 1.0356 with variances of 0.0000472 and 0.01639, respectively. Therefore, the root mean square values of *A*_0_ and *t* are 0.276 and 1.0435, respectively, and the dependence of *n* on the target strain *ε* can be expressed as

(4) $$n=0.276-A_{1}\times \exp\left[-\varepsilon /1.0435\right]$$

**Figure 8:**
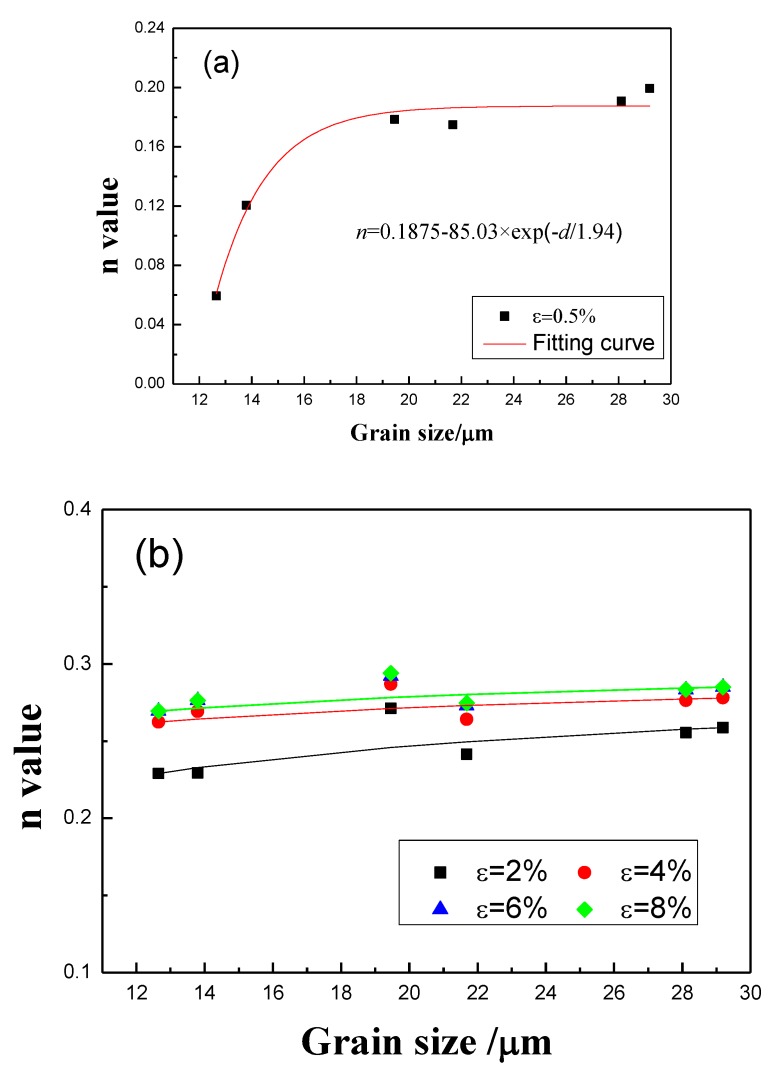

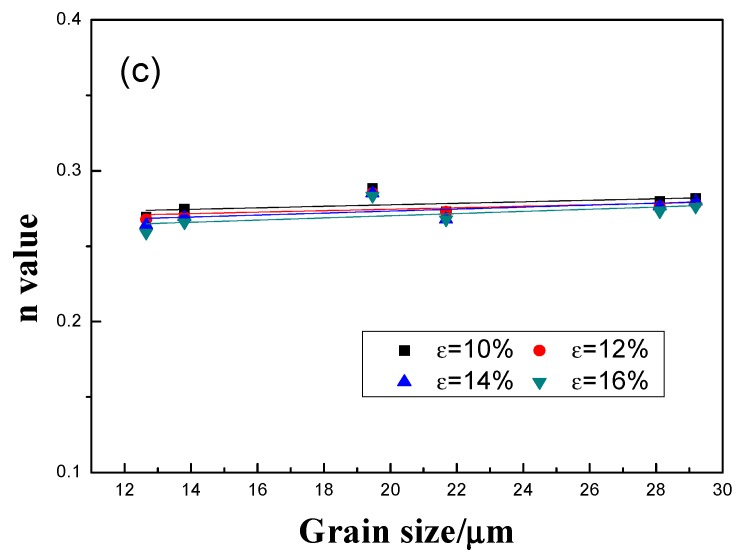
Relationship between grain size and *n* value at different target strains: (**a**) *ε* = 0.5%; (**b**) *ε* = 2%, 4%, 6%, and 8%; (**c**) *ε* = 10%, 12%, 14%, and 16%.

Figure 8 plots the dependence of *n* on the grain size for different target strains. It can be seen that the *n* value increases with the increasing of *d*, which is consistent with the result of AA1050 aluminum alloy sheet proposed by Gashti [17]. The gradient is generally positive, but the exact dependence varies with the target strain. As shown in Figure 8a, when the target strain is less than 0.5% (i.e., in the range 0.2% to 0.5%), the dependence of *n* on the grain size *d* can be expressed as:

(5) n=0.1875−85.03×exp[−d/1.94]

With increasing target strain (the target strain is greater than or equal to 2%), the relationship between *n* and *d* changes as below (see fitting results in Figure 8b,c):

(6) $$n=M-K\times d^{-1/2}$$

where both M and K are constant values summarized in Table 2. M remains approximately 0.3 for all the target strains, while K is approximately 0.15, although the target strain is 2%. Therefore, when the target strain is greater than 2%, the dependence of *n* on the grain size *d* can be expressed as:

(7) $$n=0.3-0.15d^{-1/2}$$

According to Equation (7), the relationship between *n* and the grain size in 3104 alloy sheets differs from the model established by Morrison for low-carbon steel [14], namely $n=\frac{5}{10+d^{-1/2}}$, but is similar to the model established by Qiu for ultrafine-grained steels [16], namely $n=0.3301-0.2401d^{-1/2}$ (*d* in the range of 1.30~37 μm). On the other hand, if we remove explicit references to quantities other than the grain size, Equation (1) simplifies to:

(8) $$n=0.450-0.001\times [\frac{6.6}{\sqrt{d}}+A]=C_{1}-C_{2}\times d^{-1/2}$$

where *C*_1_ and *C*_2_ are constants. Equation (7) then resembles Equation (8), in so far as *n* decreases linearly with *d*^−1/2^. However, the applicable target strain is in the range 0.1%~0.2% in Antoine’s model (Equation (1)) for Ti-IF steel, whereas, in the present work, it exceeds or equals to 2% and the grain size should be less than 29.19 μm for 3104 alloy sheets to obey Equation (6). This indicates that the relationship between *n* and the grain size differs in low-carbon steel, ultrafine-grained steels, Ti-IF steel, and 3104 alloy sheets, though *n* consistently increases with increasing grain size in all four materials. The differences are mainly attributed to the differences in crystal structure and the distributions of secondary particles in steel and aluminum alloy.

In the early stage of deformation (strain below 0.5%), as shown in Figure 8a, the *n* values for sheets with different grain sizes are relatively small, indicating the weak effect from strain hardening. With increasing strain, *n* increases rapidly with increasing grain size, followed by a slower increase or a saturation (Figure 7). Because the dislocation density is initially relatively low, grain boundaries and secondary particles are the dominant obstacles for dislocations. Although the total grain boundary area decreases with increasing grain size, flow stress also decreases (Figure 5), leading to an increase in obstacles to dislocation motion. Thus, for a given strain, the rate of strain increase is higher as a result of stronger strain hardening. This is the likely reason for the rapid increase in *n* with grain size. With further increase of strain, *n* increases more slowly, with a positive grain size dependence. This can be attributed to additional sources of obstacles to dislocation motion, e.g., the activation of multislip systems, and the formation of jogs and Lomer-Cottrell dislocation locks (arising at the intersection of dislocations under strains in excess of 2%). The proportion of obstacles associated specifically with grain boundaries is thus reduced. Therefore, the rate of increase of *n* with grain size is much lower for strains higher than 4%.

Generally, deep-drawing properties will be improved with increasing *n* value [26]. Therefore, it seems that the deep-drawing properties of studied annealed 3104 aluminum sheet can benefit from the coarser grain since the *n* increases with the grain size in the studied range (~29 um). However, an orange-peel microstructure is often observed in specimens with larger grains after deep drawing [27,28], leading to an inferior surface finish of the final product. Further study is therefore necessary to find the best balance between *n* and the grain size, in order to improve the deep-drawing properties and the quality of the surface finish.

## 4. Conclusions

(1) When increasing grain size, similar serrated flow phenomena (also known as the Portevin-Le Chatelier (PLC) effect) were observed under all conditions in studied annealed 3104 aluminum sheet. However, the yield strength (*R_p0.2_*) decreased significantly, which followed the Hall-Petch equation ($R_{p0.2}=36.273+139.8d^{-1/2}$) in the studied range of grain size (12.56–29.19 μm).
(2) The relationship between *n* and the target strain *ε* obeyed $n=0.276-A_{1}\times \exp\left[-\varepsilon /1.0435\right]$, where *A*_1_ is varying with *d* and its value is in the range of 0.132–0.364.
(3) Generally, *n* increases with grain size *d* but it follows different tendencies with *d* during the various range of strain. When the strain is 0.2–0.5%, the relationship is: $n=0.1875-85.03\times \exp{\left[-d/1.94\right]}^{}$, while it changes to $n=0.3-0.15d^{-1/2}$ when the strain is greater than 2%.

## Figures and Tables

**Table 1:** Parameters in Equation (3) under various grain sizes.

Parameters in Equation (3)	Grain Size/μm					
	12.65	13.8	16.46	21.68	28.11	29.19
*A*_0_	0.26818	0.27149	0.28778	0.27042	0.28061	0.27934
*A*_1_	0.36385	0.23346	0.20038	0.14191	0.13157	0.14068
*t*	0.91024	1.11051	0.82007	1.19239	1.08674	1.09368

**Table 2:** Parameters in Equation (6) for the *n* value at different target strains.

Target Strain	2%	4%	6%	8%	10%	12%	14%	16%
Parameter in Equation (6)								
M	0.3160	0.3081	0.3151	0.3148	0.3057	0.2976	0.3100	0.3097
K	0.3091	0.1623	0.1624	0.1613	0.1291	0.1062	0.1634	0.1811
